# Continued Breastfeeding in a Birth Cohort in the Western Amazon of Brazil: Risk of Interruption and Associated Factors

**DOI:** 10.3390/nu16193408

**Published:** 2024-10-08

**Authors:** Déborah de Oliveira Togneri Pastro, Fernanda Andrade Martins, Alanderson Alves Ramalho, Andreia Moreira de Andrade, Simone Perufo Opitz, Rosalina Jorge Koifman, Ilce Ferreira da Silva

**Affiliations:** 1 Graduate Program of Community Health, Center of Health and Sports Science, Federal University of Acre, Rio Branco 69920-900, AC, Brazil; debytog.pastro@gmail.com (D.d.O.T.P.); alanderson.ramalho@ufac.br (A.A.R.); andreia.andrade@ufac.br (A.M.d.A.); simone.opitz@ufac.br (S.P.O.); rosalina.koifman@hotmail.com (R.J.K.); ilce23@hotmail.com (I.F.d.S.); 2 Department of Epidemiology and Quantitative Methods in Health, National School of Public Health Sérgio Auroca, Fundação Oswaldo Cruz, Rio de Janeiro 21041-210, RJ, Brazil

**Keywords:** continued breastfeeding cessation, associated factors, child nutrition, actuarial analysis

## Abstract

Background: Continued breastfeeding reduces infant mortality and provides nutritional, immunological, and developmental benefits for the child. Objectives: A prospective cohort study conducted in 2015 followed 608 children who were breastfed between 6 and 24 months. The study assessed the risk of breastfeeding interruption at 12, 18, and 24 months, as well as the factors associated with this outcome, in a cohort of newborns in Rio Branco, using the life table method. Methods: The factors associated with breastfeeding cessation and their 95% confidence intervals (CI95%) were analyzed using both crude and adjusted Cox proportional hazards regression in a hierarchical model. The risks of breastfeeding cessation at 12, 18, and 24 months were 19%, 65%, and 71%, respectively. Results: Factors positively associated with the risk of breastfeeding cessation include the use of a pacifier before 6 weeks of age (HR = 1.62; CI: 95% 1.24–2.11) and the use of a bottle during the first year of life (HR = 1.41; CI: 95% 1.11–1.78). Maternal return to work after the birth of the baby (HR = 0.78; CI: 95% 0.62–0.97) was found to be negatively associated with the risk of breastfeeding interruption. Conclusions: Early pacifier use before 6 weeks and the introduction of a bottle in the first year affect continued breastfeeding. Maternal employment was associated with reduced risk of breastfeeding cessation, contrary to most studies.

## 1. Introduction

Infant mortality still represents a serious public health issue, especially in developing countries [1]. In Brazil, the infant mortality rate has declined in recent years, decreasing from 47.1 per 1000 live births (LB) in 1990 to 13.3 per 1000 LB in 2019. A similar decline was observed in the northern region during the same period, from 45.9 to 16.6 per 1000 LB [2]. Among the main strategies recommended by the World Health Organization (WHO) and the Brazilian Ministry of Health (MH) to reduce infant and under-5 mortality is exclusive breastfeeding (EBF) until 6 months of age and continued breastfeeding (CBF) until 24 months of age [2,3].

Although the benefits of EBF are well established, there is currently increasing evidence highlighting the benefits of breastfeeding from 6 to 24 months of age [4,5]. The benefits of CBF include a 50% reduction in the risk of mortality from infectious diseases, a 30% reduction in the risk of diarrhea in children under 5 years of age, and a 32% reduction in the risk of hospitalizations due to respiratory diseases in children under 2 years of age [6,7,8].

Breast milk (BM) contributes to infant nutrition as an important source of nutrients, providing approximately half of the energy needs in children aged 6 to 12 months and one-third of the energy needs in infants aged 12 to 24 months [9]. The benefits of BM show a dose–response relationship with the frequency and duration of breastfeeding. Despite the decline in the level of immunoglobulin A in BM during breastfeeding, its biological activity in inhibiting bacterial adhesion remains unchanged, providing protection against gastroenteritis throughout the breastfeeding period [6,10].

Despite the extensive knowledge accumulated regarding the benefits of CBF, the frequency of this practice is still below expectations. A study that evaluated the global prevalence of WHO-recommended feeding practices in 57 low- and middle-income countries showed that the prevalence of CBF up to one year of age and up to two years of age was a respective 87.2% and 50.7% in Africa (33 countries), 70.4% and 40.6% in the Americas (6 countries), 72.2% and 38.7% in the eastern Mediterranean (5 countries), 66% and 34.8% in Europe (4 countries), and 84.8% and 69.3% in southeast Asia/western Pacific (9 countries) from 2010 to 2018 [11].

In Brazil, a temporal trend study using secondary data from national population-based surveys conducted in 1986, 1996, 2006, and 2013 revealed that the prevalence of CBF up to 2 years of age was approximately 25% between 1986 and 2006, increasing to 31.8% in 2013 [12]. Despite this increase over time, the National Study of Child Feeding and Nutrition (ENANI), conducted between 2019 and 2020, found that the national prevalence of BF in children aged 20 to 23 months is still considered low (35.5%), with higher rates in the northeast region (48%), followed by the south (42.7%), north (39%), midwest (32.3%), and southeast (23.4%) regions [13].

However, few studies have evaluated the risk of continued breastfeeding cessation and its associated factors. Maternal age under 25 years, maternal return to work before the child reaches 6 months of age [14], and lack of exclusive breastfeeding in the first 6 months of life [15] have been associated with breastfeeding cessation before the age of two in international studies. In the rural areas of Acre state, Brazil, the average duration of CBF was 16.7 months and was associated with the delayed initiation of breastfeeding within the first hour of life, with pacifier and bottle use, and with previous breastfeeding experience of less than 6 months. In the same study, the likelihood of a child being breastfed at 24 months was 49.7% [16].

Despite this, the primary studies on CBF conducted so far have had limitations, such as small sample size [15], significant loss to follow-up [14], and a lack of the information regarding the reasons for loss to follow-up that is needed to assess the probability of selection bias due to non-random loss to follow-up [16]. Rio Branco, the capital of Acre in the northern region of Brazil, is characterized by a rich cultural diversity influenced by both indigenous and non-indigenous traditions, as well as by challenging socioeconomic conditions due to the low income of the local population. Its geography, with extensive forested areas, limits access and affects the availability of healthcare services [17]. Given the scarcity of information on the prevalence of CBF from 6 to 24 months and associated factors, especially in the northern region of the country, the present study aimed to estimate the risk of breastfeeding interruption from 6 to 24 months and associated factors in a cohort of live births from 2015 in Rio Branco, Acre.

## 2. Materials and Methods

This is a prospective study of a birth cohort that included subjects born between April and June 2015 in Rio Branco, Acre, who were followed up with until 24 months of age. The study integrates two parent projects: “Evolution of nutritional indicators from birth to the first year of life in Rio Branco, Acre” and “Breastfeeding, early weaning, minimum acceptable diet, and overweight in a cohort of live births in 2015, Rio Branco-AC” [18,19]. All stages of the study were conducted in accordance with Resolution 466/12 of the National Health Council, approved by the Research Ethics Committee of the Federal University of Acre, under CAAE: 40584115.0.0000.5010 and 57135516.8.00005240. The sample size of the 2015 main birth cohort has been previously published [18,19]. In summary, considering a population base of 6965 live births in 2015, where the mothers resided in the city of Rio Branco, AC; a prevalence of breastfeeding in the first hour of life of 50%; a precision of 0.05; a confidence interval of 95%; a study power of 80%; and an OR of 2.0. The estimated sample size was 964 live births. The sample was inflated by 10% to reduce the non-response effect, comprising a sample size of 1060 live births.

**Figure 1:**
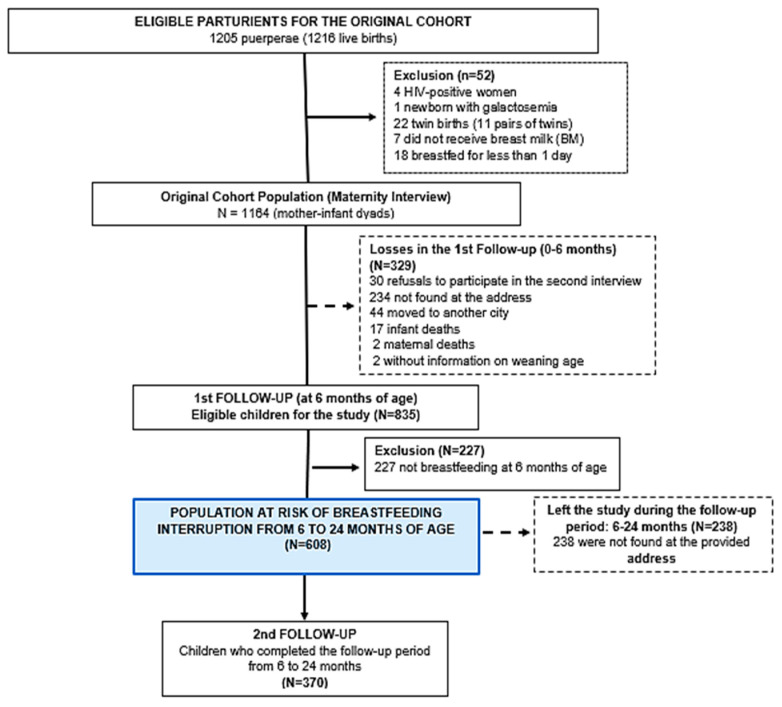
Flowchart of participants in the birth cohort study “Evolution of Nutritional Indicators from Birth to the First Year of Life in Rio Branco, Acre,” for the analysis of factors associated with continued breastfeeding cessation up to 2 years of age in a cohort of live births in Rio Branco, Acre.

The major cohort follow up dynamic from birth to 6 months old, and the breastfeeding information up to 6 months have already been explored and have been published previously [18,19]. Shortly, among the 1216 children initially included in the 2015 birth cohort, 52 (4.2%) were excluded because they were born to mothers with HIV (n = 4), had galactosemia (n = 1), were twins (n = 22), did not receive breast milk (n = 7), or breastfed for less than 1 day (n = 18). Of the 1189 children remaining in the study, 329 (27.7%) were lost to follow-up from 0 to 6 months. However, these losses were considered random, as there were no statistically significant differences between the lost children and those who remained in the study regarding sociodemographic and birth variables [18]. Among the 835 children followed up to six months of age, 227 (27.2%) had discontinued breastfeeding. During the follow-up period from birth to six months, the sociodemographic characteristics, birth profiles, and hospital breastfeeding characteristics of the children who were lost to follow-up were statistically similar to those of the children who continued in the cohort [18,19]. For the analysis of the risk of continued breastfeeding cessation (CBF), 608 children who were still breastfeeding at 6 months old and therefore were at risk of discontinuing breastfeeding between 6 and 24 months were considered. Of these 608 children, 238 (39.1%) were lost to follow-up as they could not be located at the addresses provided at the start of the study. Therefore, the losses contributed with half of their follow-up time to meet the life-table analysis assumptions. Thus, 370 children completed the 24 months of follow-up (Figure 1).

The data collection was conducted by a team of health sciences students, who were trained and supervised by researchers from the Federal University of Acre [18,19]. They used questionnaires developed by the research team, from which the variables analyzed in the study were constructed. Entry interviews were conducted within the first 48 h postpartum, and follow-up visits were conducted at participants’ homes, scheduled in advance via telephone. In cases where phone contact was unsuccessful, active search at the provided addresses was conducted. If participants were not available during the scheduled home visit, additional visits were made at different times, including weekends (an average of three visits per household).

The independent variables obtained through interviews and confirmed directly from the mother’s or child’s medical records, birth certificate, or pregnancy card were as follows: age (≤19, 20–34, or ≥35 years); skin color (white, brown, or other); maternal education (up to 8 years or more than 8 years); marital status (with or without a partner); number of living children (1, 2 or 3, or 4 or more); smoking during pregnancy (yes or no); number of prenatal visits (<6 or ≥6 visits); birth history (primiparous or multiparous); prematurity (yes or no); baby’s sex (male or female); and prenatal care sector (public or private), defined by the highest number of prenatal visits.

The following variables were obtained exclusively through interviews conducted at the maternity ward: income categorized according to the minimum wage (MW) at the time (≤1 MW, 1 to 3 MW, or >3 MW); beneficiary of the Bolsa Família social assistance program (yes or no); socioeconomic class defined according to criteria from the Brazilian Association of Research Companies (A and B or C, D, and E); planned pregnancy (yes or no); counseling on breastfeeding during prenatal care (yes or no); alcohol and cigarette consumption during pregnancy (yes or no); cross-nursing (breastfeeding another woman’s child yes or no); assistance for breastfeeding in the maternity ward (yes or no); mother’s desire for breastfeeding duration (<6, 6 or >6 months); and self-reported postpartum depression (yes or no). The Edinburgh Postnatal Depression Scale was used to assess symptoms of postpartum depression [20]. The EPDS is a 10-item scale, with four possible answers for each question (scoring from 0 to 3 each). These questions assess the presence and intensity of depression symptoms experienced in the past 7 days. The sum of EPDS scores may vary from 0 to 30 points [20]. The EPDS was validated for the Brazilian population in 2009 [21] and the cut-off point for the presence of depression symptoms was a score > 10. However, in the present study, none of the participants scored 10+ points, limiting the analysis of this variable.

During the follow-up interview, information was obtained regarding maternal work interruption after the baby’s birth (yes or no), age of initiation of childcare follow-up (<7 days or ≥7 days), introduction of complementary feeding before six months (yes or no), use of bottle in the first year of life (yes or no), use of a pacifier before 6 weeks old (never used/used ≤6 weeks/used >6 weeks), and paternal involvement in breastfeeding (yes or no). Breastfeeding practices were assessed at hospital discharge and during follow-up, based on WHO definitions [3]. Children who were exclusively breastfeeding at hospital discharge were classified as exclusive breastfeeding (EBF). Those who were exclusively breastfeeding at discharge but had received formula only during their hospital stay were classified as EBF with early formula use (EBFme). Children who were receiving both breast milk and formula at discharge were classified as mixed breastfeeding (MBF). This study also included babies who were breastfed by other women. We also investigated whether cross-nursing practices were used, in which infants were fed with another woman’s milk.

**Figure 2:**
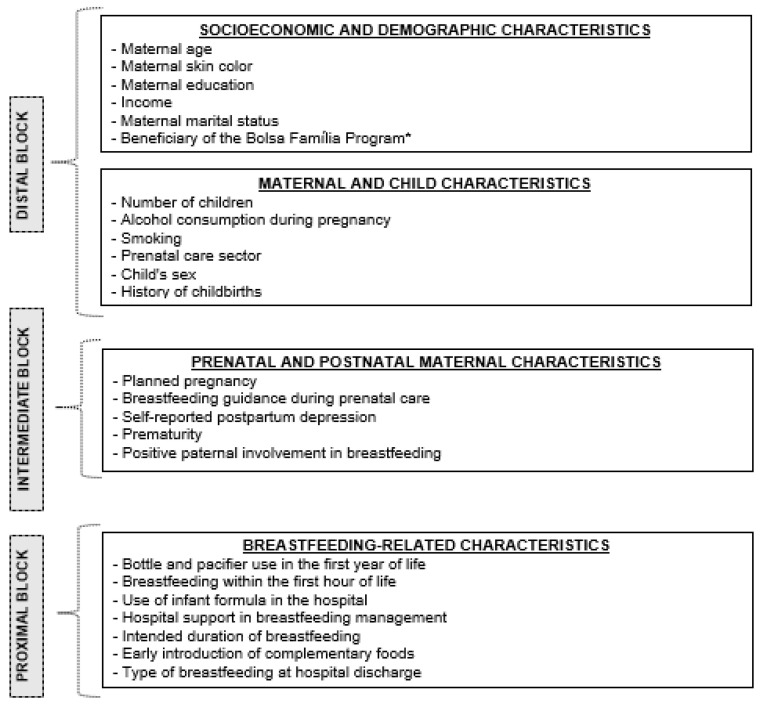
Hierarchical theoretical model with the independent variables. * The Bolsa Família program (BFP) is a federal program of direct and indirect income transfer.

A hierarchical theoretical model was developed in which the independent variables were grouped into blocks according to their relationship with the outcome. In the approach adopted [22], the blocks were organized hierarchically based on the proximity of each exposure factor to the outcome. The different variables were distributed into three blocks. The first block (distal) comprised socioeconomic and demographic variables and maternal and child characteristics, the second block (intermediate) included maternal characteristics from the prenatal and postnatal periods, and the third block (proximal) encompassed variables related to breastfeeding (Figure 2).

The outcome variable of breastfeeding cessation at 12, 18, and 24 months of age was defined by the question “At what age did the child stop breastfeeding?” obtained during the follow-up interview.

Participant characteristics were described using means (standard deviations, SD) for continuous variables and proportions (%) for categorical variables. Differences between proportions of categorical variables were assessed using Pearson’s chi-square test and Fisher’s exact test (5% significance level), while differences between means were assessed using the Student’s *t*-test (for normally distributed variables) and differences between medians were assessed using the Mann–Whitney U test (for variables with non-normal distribution).

To estimate the risk of continued breastfeeding cessation at 12, 18, and 24 months of age, a survival function for each independent variable, we used the actuarial life table method. Failure was defined as discontinuation of continued breastfeeding (weaning), while censoring was defined as loss to follow-up of individuals or the end of the study period. The follow-up time was defined as the days elapsed between 6 months of age and either failure or censoring. Exact dates of loss to follow-up were not available; therefore, it was assumed that losses contributed half of the follow-up time between interviews. However, losses occurred randomly, and there were no statistically significant differences in sociodemographic and birth variables between children lost to follow-up and those who remained in the study. Differences between survival curves were evaluated using the Wilcoxon (Gehan) test, with statistically significant differences defined as those with a *p*-value less than 0.05.

The magnitudes of associations between the independent variables and breastfeeding cessation, along with their 95% confidence intervals (CI), were estimated using the proportional Cox regression model, and statistical significance was assessed by the Wald test. Subsequently, a hierarchical conceptual model was adopted. Collinearity among variables within blocks was tested, and variables with correlations greater than 0.5 were removed from the conceptual model. Independent variables with a *p*-value < 0.20 in the crude HR analysis were selected for testing in multiple models, with multiple analyses conducted from the distal to proximal block. Within each block, variables were introduced simultaneously, and those that did not show statistical significance (*p* < 0.05) in the Wald test were individually removed, respecting the descending order of *p*-values. Variables whose removal caused a change of more than 10.0% in the magnitude of hazard ratios (HR) of the block variables were retained in the model.

Statistical analyses were performed using the Statistical Package for the Social Sciences version 26.0 (SPSS 26.0).

## 3. Results

Of the 608 mother–infant pairs included in the study, 65.6% of the mothers were aged 20 to 34 years, indicating a predominance of mothers in the productive and young-adult age group. Most, 84.5%, identified as brown (mixed-race). In terms of education, 73.8% of the mothers had more than 8 years of schooling, reflecting a relatively high educational level for this population, especially considering that 21.1% of them had a monthly family income below the minimum wage. The family structure appears to be relatively stable, as 85.5% of the mothers lived with a partner. Additionally, 92.5% of the mothers were non-smokers, and 84.4% reported not consuming alcoholic beverages, indicating positive health behaviors (Table 1).

Throughout the follow-up period from 6 to 24 months, 238 (39.1%) children were not found at the reported address (Figure 1). The risk of continued breastfeeding interruption was 19%, 65%, and 71% at 12 months, 18 months, and 24 months, respectively. This gradual increase suggests a trend toward greater difficulty in maintaining continued breastfeeding as the child grows. The risk of continued breastfeeding interruption at 24 months was 81% for mothers who consumed alcohol (*p* = 0.004), 67% for those who did not stop working due to the baby’s birth (*p* = 0.005), 81% for children who used a bottle in the first year of life (*p* = 0.001), and 83% for those who used a pacifier in the first year of life (*p* < 0.000), indicating that all of these factors may be associated with a higher likelihood of breastfeeding cessation (Table 2).

**Figure 3:**
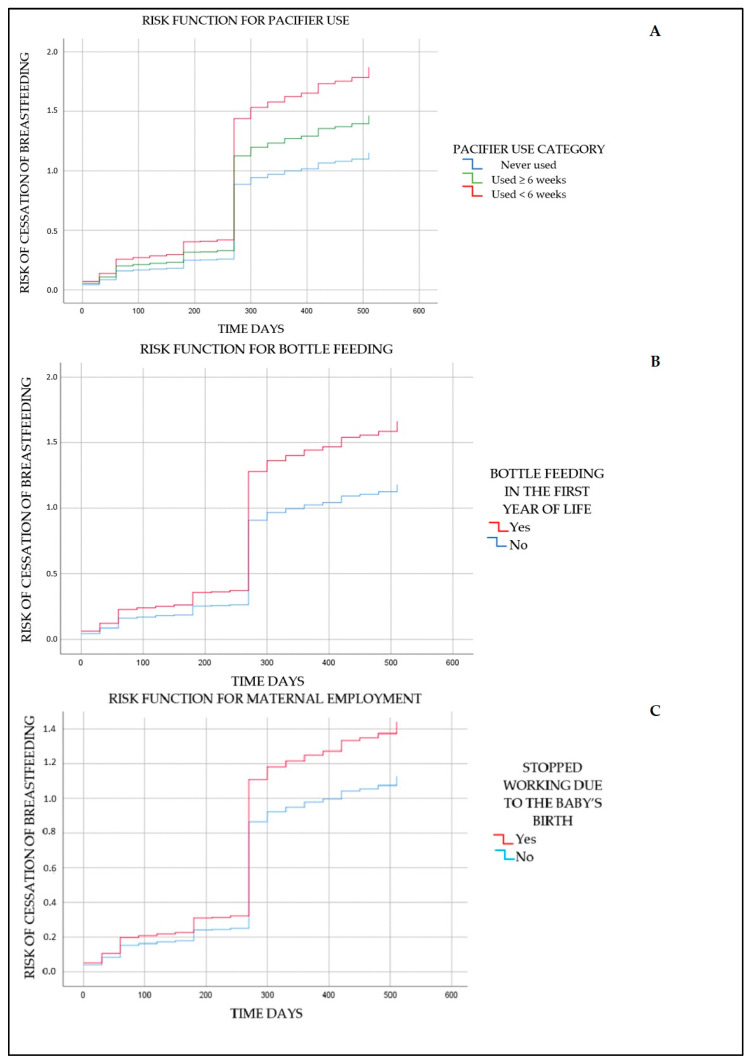
Risk functions for continued breastfeeding cessation from 6 to 24 months in a cohort of children from the Brazilian western Amazon. (**A**) Risk function for pacifier use; (**B**) risk function for bottle feeding; (**C**) risk function for maternal employment cessation after the baby’s birth.

In the hierarchical analysis, it was observed that factors positively associated with the risk of continued breastfeeding interruption between 6 and 24 months of age included pacifier use before 6 weeks of age (HR: 1.62; 95% CI: 1.24–2.11) and bottle feeding in the first year of life (HR: 1.41; 95% CI: 1.11–1.78). On the other hand, the factor negatively associated with the risk of weaning was the mother stopping work after the baby’s birth (HR: 0.78; 95% CI: 0.62–0.97), a fact that contradicts the majority of studies (Table 3 and Figure 3).

## 4. Discussion

In Rio Branco, Acre, the median duration CBF was 270 days, a value similar to that observed in the ENANI study conducted in 123 Brazilian municipalities (CBF = 300 days) [13], and in cohort studies conducted in Sichuan, China (CBF = 240 days) [14], and Australia (CBF = 200 days) [23]. These results indicate that the observed median durations of CBF are lower than the World Health Organization (WHO) recommendation of 540 days or more [4].

Despite the duration of CBF in Rio Branco being similar to that found in the ENANI study, which may reflect the reality of CBF duration in Brazil, its median CBF was still 30 days longer than in China and 70 days longer than in Australia. One possible explanation for this difference compared with the Australian study could be easier access to infant formulas in that country. In the case of China, the traditional consumption of teas and their early introduction to children may explain this difference.

In this regard, in Rio Branco, there was a gradual increase in the risk of continued breastfeeding cessation from 6 to 24 months, ranging from 19% at 12 months to 71% at 24 months, respectively. This risk of breastfeeding cessation can be explained by the child’s increasing dependence on maternal care up to 12 months, while, during the second year of life, there is a progressive gain in the child’s independence [23]. On the other hand, starting at 12 months, many children begin attending daycare centers and schools, which can facilitate weaning before reaching 24 months of age [24].

The conditional probability of breastfeeding interruption by 24 months in Rio Branco was found to be similar to that observed in a cohort study conducted in Feira de Santana, Bahia (weaning risk = 79.2%) [25], and higher than that observed at 12 and 24 months in Indonesia (10% and 51%, respectively) [15]. Indonesia is known for its robust public policies promoting exclusive and continued breastfeeding due to high rates of malnutrition. Additionally, the country has a strong market for promoting infant formula use. These circumstances may explain the differences observed in CBF rates at 24 months compared with the present study [26].

Factors associated with weaning before 6 months are well-documented in the literature [27,28]. In this cohort of live births from Rio Branco, Acre—a region with few studies on these aspects—it was possible to evaluate important maternal and infant outcomes related to breastfeeding, such as the influence of cesarean delivery and low birth weight on breastfeeding within the first hour of life, as well as the importance of exclusive breastfeeding at hospital discharge for reducing the likelihood of weaning before six months [18,19]. On the other hand, factors associated with the risk of the interruption of breastfeeding from 6 to 24 months may involve various maternal and sociopolitical elements that can differ from population to population or among groups within the same population [26,29]. In the present study, the use of pacifiers before 6 weeks of age and bottles in the first year of life were found to be positively associated with the risk of breastfeeding interruption from 6 to 24 months of age.

The association between pacifier use and breastfeeding interruption is consistent with a meta-analysis showing a strong link between the introduction of pacifiers before 6 weeks of age and weaning before 6 months, as well as between 6 and 24 months of age. [30]. National studies have also observed an elevated risk of breastfeeding interruption at 24 months of age associated with pacifier use, as seen in Porto Alegre, Rio Grande do Sul (PR: 1.29; 95% CI: 1.07–1.54) and Guarapuava, Paraná (PR: 1.47; 95% CI: 1.30–1.66) [31,32]. The use of pacifiers is a cultural practice in the north region of Brazil, promoting non-nutritive sucking and interfering with breast milk production [18]. Regarding bottle use, a cross-sectional study conducted during the first year of life in a birth cohort in Cruzeiro do Sul, Acre, found an inverse association between bottle use and continued breastfeeding, highlighting a 40% increased risk of weaning from complementary breastfeeding in children who used bottles (PR: 1.44; 95% CI: 1.33–1.57) [16]. Similarly, in Guarapuava, Paraná, this probability was approximately five times higher among children who used this device (PR: 4.74; 95% CI: 3.45–6.52) [32]. Unfortunately, the questionnaire used in this study did not include information on bottle use before 6 months, limiting the analysis. Despite this, we found a strong association (HR = 1.41; 95% CI: 1.11–1.78) between bottle use in the first year of life and weaning between 6 and 24 months of age, independent of pacifier use and the absence of maternal work interruption.

In this sense, international studies support the influence of bottle use on the cessation of CBF. A cohort study conducted in China observed that both children who used bottles before six months of age (OR: 2.40; 95% CI: 1.80–3.21) and those who used bottles after six months (OR: 2.28; 95% CI: 1.59–3.28) had similar odds of weaning. This suggests that the risk of CBF cessation is high regardless of the age at which bottles are introduced [33].

There are numerous reasons why mothers opt for bottle feeding when they start CBF, one of the most significant being their return to work after maternity leave, which in Brazil extends to 6 months after childbirth [14,34]. Consequently, the use of bottles becomes necessary to supplement feeding, potentially leading to the child losing interest in breastfeeding. These factors may explain the association found between bottle use and the reduction in breastfeeding up to the second year of life, as observed in this study.

The success of continued breastfeeding beyond 6 months of age depends not only on the mother but also on a combination of factors and interventions involving the family, healthcare services, supportive public policies, and conditions for returning to work that ensure the continuation of breastfeeding [35,36]. In addition to quantitative studies, a qualitative study conducted in Turkey with Syrian refugees, using structured discussions with Turkish and Syrian healthcare professionals, pregnant Syrian refugees, mothers, fathers, and grandmothers, found that most Syrian healthcare workers had not received training in breastfeeding counseling. The short duration of breastfeeding among Syrian refugees was linked to cultural characteristics and migration. Some cultural characteristics can be summarized as “believing that breastfeeding harms the mother’s health;” “teen marriages;” “desiring to have as many children as possible;” “giving anise to infants and not breastfeeding at night;” “pre-lacteal feeding;” “believing that milk is not enough;” “excessive control of mother–child interaction by grandmothers, which limits interaction;” “short intervals between pregnancies;” and “not using modern family planning techniques” [36].

Surprisingly, maternal return to work after childbirth was found to be another factor statistically associated with the cessation of exclusive breastfeeding (EBF) in this study, reducing the risk of continued breastfeeding by 22%. Contrary to what has been observed in national studies [25,35] and some international studies [26,37], this study found a negative association between maternal employment after childbirth and the risk of EBF. Although counterintuitive, this result is similar to a cross-sectional study conducted in Timor-Leste, which found that the continuation of breastfeeding at the end of the first year was significantly lower among mothers who did not work (OR: 1.58; 95% CI: 1.10–2.27) compared with those who worked [38]. Both in our study and in the Timor-Leste study, this association warrants careful evaluation, as it is plausible that maternal employment negatively impacts continued breastfeeding due to the physical separation between mother and child, emotional impact related to returning to work, and the need to introduce new foods [23,39].

A possible explanation for the association between maternal employment and a lower risk of continued breastfeeding cessation in our population could be that most women received low incomes and might hold informal or temporary jobs. Mothers working in the informal sector often face greater economic instability and lack of labor benefits, which can result in financial stress and insecurity [40]. Maternal employment emerges as a significant factor in breastfeeding cessation, as some mothers choose to replace breast milk with commercial alternatives upon returning to work after maternity leave, leading to complete cessation of breastfeeding [41]. On the other hand, incentives and policies that support continued breastfeeding can alter this trajectory. An example of this was observed in a survey conducted in a private company in Taiwan, which receives government financial support to implement internal breastfeeding promotion policies. Women with access to breastfeeding facilities at work showed a higher intention to breastfeed for more than 6 months after returning from maternity leave (OR: 5.33; 95% CI: 1.98–14.3) [42].

In Brazil, maternity leave is 120 days from the birth of the baby, and it can be extended up to 180 days through a voluntary adherence program called “Empresa Cidadã” (Citizen Company) [43]. Despite advances in the country regarding the extension of maternity leave, this measure does not yet guarantee the continuity of breastfeeding beyond 180 days, as most companies lack adequate facilities such as equipped rooms for expressing and storing breast milk. The increase in female participation in the labor market, the short duration of maternity leave, and the lack of extended labor rights beyond the sixth month of the child’s life significantly hinder the continuation of breastfeeding until two years of age [41,44].

The literature supports the idea that a mother’s choice regarding breastfeeding and its continuation after 6 months is influenced by an intersection of sociodemographic, psychological, and cultural factors. These factors may include younger age, lower educational levels, lack of confidence in the nutritional power of breast milk, family pressure to supplement breastfeeding, emotional insecurity, and insufficient professional support for breastfeeding [36,45,46,47,48]. Regarding continued breastfeeding from 6 to 24 months, support from healthcare professionals, maternal age, and lower education levels did not remain in the final model (Appendix B). On the other hand, after adjusting for maternal age, it was found that child’s sex, maternal alcohol use, the prenatal care sector, pacifier and bottle use, and maternal employment each strongly affected the risk of weaning between 6 and 24 months. These results support the idea that continued breastfeeding requires promotion and engagement on multiple fronts. This includes education, providing detailed information on the numerous benefits of continued breastfeeding, as well as offering technical support to help mothers overcome common breastfeeding challenges [14,37,49]. Additionally, guidance is needed to develop breastfeeding strategies tailored to individual routines and needs [50,51]. Therefore, public policies that promote breastfeeding should ideally start during prenatal care and continue postpartum, ensuring support and guidance for mothers to reduce the inherent difficulties of breastfeeding [35].

Although positive paternal involvement in breastfeeding support was inversely associated with the risk of weaning before 2 years, this factor did not remain in the final model of the present study. While positive paternal involvement in breastfeeding support is strongly associated with breastfeeding from 0–6 months [22,35,51,52], for breastfeeding from 6–24 months, the effect of paternal involvement tends to be around 18% [52]. Therefore, in the presence of factors with higher magnitude associations, such as pacifier and bottle use, as observed in this study, the effect of paternal involvement in breastfeeding support was not strong enough to remain in the final model.

Despite maternal age being associated with breastfeeding up to 6 months [29,52], for continued breastfeeding, the associations are around 2% for each year of maternal age [49]. In the present study, although the magnitude of the association with maternal age was 2% for each year of maternal age, no statistical significance was observed for this condition. This lack of significance may be attributed to the sample size of the present study, which is considered small for achieving statistical significance. Younger women tend to breastfeed less frequently due to various factors, such as lack of experience, lower understanding of the importance of breastfeeding, and limited partner support. In contrast, older women, who often have higher educational levels and financial stability, may be more successful in maintaining prolonged breastfeeding.

To the best of our knowledge, this study was the first population-based prospective study aimed at determining factors associated with continued breastfeeding (CBF) interruption in the western Amazon region of Brazil. The use of a longitudinal cohort design allowed for us to track children over a critical period for breastfeeding, from 6 to 24 months. Another notable aspect is the application of a hierarchical model in the data analysis, which enabled the evaluation of the effect of each variable according to its importance and directionality, supporting the causal relationship between the analyzed exposures and breastfeeding interruption. The findings provide valuable evidence that can inform public health strategies and policies to promote continued breastfeeding, especially in low-income and high-vulnerability contexts, such as those found in the studied region.

The present study has limitations that must be addressed. First, the present study presented 39.1% of follow up, as some of the subjects had moved to another address or city. Such a loss reflects the residential instability in the region, stemming from the low income and high social vulnerability of the local population, who lived in temporary rentals. However, this loss was not statistically different from those who remained in the study concerning sociodemographic and birth variables. Therefore, the losses were randomly distributed, reducing the risk of selection bias (as detailed in Appendix A). Thus, it is likely that the risk of outcomes in the lost-to-follow-up group is similar to that of the group that continued in the study. Additionally, there is a possibility of changes over time in the socioeconomic status, such as socioeconomic classification (according to ABEP), income, education level, family benefits, and marital status. However, given the short time between the interviews (up to 24 months), it is highly likely that these changes occurred similarly for both participants who weaned and those who continued breastfeeding from 6 to 24 months. This could have led to non-differential misclassification, which tends to underestimate associations, bringing them closer to 1. If, despite this, we could observe associations between exposure and outcome, this means that, in reality, the magnitudes of such associations would be even stronger if such a to non-differential misclassification did not exist. In addition, significant and substantial associations were found with all traditionally established risk factors for breastfeeding interruption between 6 and 24 months, such as the use of pacifiers and bottles and maternal return to work, corroborating with existent evidence regarding factors associated with breastfeeding interruption from 6 to 24 months. To improve the understanding of the factors contributing to the interruption of continued breastfeeding in the Amazon region, it is essential for future studies to implement strategies to minimize loss to follow-up. The integration of remote monitoring technologies can facilitate continuous updates to communication channels with participants. Additionally, it is crucial to encourage research that explores the effects of changes in the socioeconomic status of Amazonian communities on the practice of continued breastfeeding. These efforts will help to elucidate the complex dynamics influencing breastfeeding in the region and to develop more effective interventions. Lastly, the lack of information on subsequent pregnancies and intervals between births also represents a limitation when analyzing an important factor that could affect weaning between 6 and 24 months. Although access to family planning methods in Brazil helps reduce the frequency of short intervals between pregnancies, this variable still needs to be investigated in future studies in western Brazilian Amazon.

Thus, given the aim of overcoming the challenges associated with ensuring continued breastfeeding until the second year of life, the implementation of awareness campaigns about the importance of sustained breastfeeding and of policies supporting breastfeeding in the workplace becomes necessary. Additionally, actions are needed to integrate health services with the social support network for mothers to provide an encouraging and supportive environment. To advance this goal, creating breastfeeding support groups through lactation consultancy and expanding training for healthcare professionals with comprehensive knowledge about breastfeeding are fundamental. Furthermore, offering flexible parental leave policies and establishing community breastfeeding support programs will contribute to maintaining breastfeeding until the child’s second year.

## 5. Conclusions

The present study observed an increased frequency of continued breastfeeding interruption in Brazilian western Amazon, reflecting low adherence to this practice. Sociocultural and economic factors, such as the early use of pacifiers and bottles, along with a lack of knowledge about the benefits of continued breastfeeding, were found to be positively associated. Furthermore, there is a complex relationship between maternal employment and the interruption of continued breastfeeding in the Amazon region. Economic and social vulnerabilities may modulate such a relationship as mothers face economic pressures that may force them to prioritize other needs over continued breastfeeding.

This study highlights the urgent need to develop strategies and policies encouraging continued breastfeeding. Although the data are from 2015, the results remain relevant, as public policies in Brazil have focused on exclusive breastfeeding, neglecting interventions for breastfeeding from 6 to 24 months. Therefore, implementing policies that support the balance between work and continued breastfeeding is crucial, as well as intensifying educational campaigns about the benefits of continued breastfeeding. However, further longitudinal studies are needed to investigate other factors influencing this practice in this region. These actions are essential for improving continued breastfeeding rates and child health in the western Amazon.

## Figures and Tables

**Table 1:** Distribution of characteristics related to the socioeconomic and obstetric conditions of the mother–infant dyads participating in the 2015–2017 cohort study (n = 608).

Variable	Total ^1^	24 Month Follow-Up	Follow-Up Losses	*X*^2^—Test
	N = 608 (100%)	N = 370 (%)	N = 238 (%)	*p*-Values
Age				0.487
≤19	122 (20.1) *	73 (59.8)	49 (40.2)	
20–34	399 (65.6) *	239 (59.9)	160 (40.1)	
≥35	87 (14.3) *	58 (66.7)	29 (33.3)	
Skin color				0.015
White	54 (8.9) *	31 (57.4)	23 (42.6)	
Brown	514 (84.5) *	323 (62.8)	191 (37.2)	
Others	40 (6.6) *	16 (40.0)	24 (60.0)	
Maternal education				0.065
Up to 8 years	159 (26.2) *	87 (54.7)	72 (45.3)	
8 years or more	449 (73.8) *	283 (63.0)	166 (37.0)	
Marital status				0.577
Without partner	88 (14.5) *	56 (63.6)	32 (36.4)	
With partner	519 (85.5) *	314 (60.5)	205 (39.5)	
Income level ^2^				0.352
≤1 MW	117 (21.1) *	66 (56.4)	51 (43.6)	
1 to 3 MW	313 (56.5) *	187 (59.7)	126 (40.3)	
>3 MW	124 (22.4) *	81 (65.3)	43 (34.7)	
Bolsa Família Program				0.847
Yes	153 (25.2) *	92 (60.1)	61 (39.9)	
No	454 (74.7) *	277 (61.0)	177 (39.0)	
Socioeconomic class				0.217
A and B	122 (20.2) *	80 (65.6)	42 (34.4)	
C, D and E	481 (79.8) *	286 (47.4)	195 (40.5)	
Smoking				0.872
Yes	45 (7.5) *	28 (62.2)	17 (37.8)	
No	559 (92.5) *	341 (61.0)	218 (39.0)	
Alcohol				0.044
Yes	95 (15.6) *	49 (51.6)	46 (48.4)	
No	513 (84.4) *	321 (62.6)	192 (37.4)	
Prenatal care (visits)				0.476
<6	157 (15.6) *	100 (63.7)	57 (36.3)	
≥6	435 (73.5) *	263 (60.5)	172 (39.5)	
Prenatal care sector				0.403
Public	513 (86.1) *	309 (60.2)	204 (39.8)	
Private	83 (13.9) *	54 (65.1)	29 (34.9)	
Planned pregnancy				0.209
Yes	230 (38.0) *	147 (63.9)	83 (36.1)	
No	376 (62.0) *	221 (58.8)	155 (41.2)	
Prenatal breastfeeding guidance				0.252
Yes	287 (58.1) *	181 (63.1)	106 (36.9)	
No	207 (41.9) *	120 (58.0)	87 (42.0)	
Birth history				0.359
Primiparous	236 (38.8) *	149 (63.1)	87 (36.9)	
Multiparous	372 (61.2) *	221 (59.4)	151 (40.6)	
Number of children				0.490
1	236(38.8) *	149 (63.1)	87 (36.9)	
2–3	183 (30.1) *	98 (53.6)	85 (46.4)	
≥4	189 (31.1) *	123 (65.1)	66 (34.9)	
Received formula in the maternity ward				0.319
Yes	76 (12.6) *	50 (65.8)	26 (34.2)	
No	525 (87.4) *	314 (59.8)	211 (40.2)	
Breastfeeding support in the maternity ward				0.389
Yes	441 (72.5) *	273 (61.9)	168 (38.1)	
No	167 (27.5) *	97 (58.1)	70 (41.9)	
Self-reported postpartum depression				0.624
Yes	114 (18.8) *	67 (58.8)	47 (41.2)	
No	493 (81.2) *	302 (61.3)	191 (38.7)	
Breastfeeding at hospital discharge ^3^				0.592
EBF	522 (85.9) *	314 (60.2)	208 (39.8)	
EBFme	66 (10.9) *	44 (66.7)	22 (33.3)	
MBF	20 (3.2) *	12 (60.0)	8 (40.0)	
Stopped working due to the baby’s birth				0.828
Yes	268 (47.9) *	162 (60.4)	106 (39.6)	
No	246 (52.1) *	151 (61.4)	95 (38.6)	
Mother’s desire for breastfeeding duration				0.349
<6 months	49 (8.2) *	33 (67.3)	16 (32.7)	
≥6 months	550 (91.8) *	333 (60.5)	217 (39.5)	
Baby’s sex				0.913
Female	321 (52.8) *	196 (61.1)	125 (38.9)	
Male	287 (47.2) *	174 (60.6)	113 (39.4)	
Positive paternal involvement in breastfeeding				0.147
Yes	509 (83.9) *	303 (59.5)	206 (40.5)	
No	98 (16.1) *	66 (67.3)	32 (32.7)	
Prematurity				0.800
Yes	51 (8.4) *	32 (62.7)	19 (37.3)	
No	553 (91.6) *	337 (60.9)	216 (39.1)	
Childcare follow-up				0.969
≤7 days	131 (23.3) *	80 (61.1)	51 (38.9)	
>7 days	432 (76.7) *	263 (60.9)	169 (39.1)	
Breastfeeding in the first hour of life				0.735
Yes	359 (60.4) *	215 (59.9)	144 (40.1)	
No	235 (39.6) *	144 (61.3)	91 (38.7)	
Cross-nursing				0.674
Yes	115 (18.9) *	68 (59.1)	47 (40.9)	
No	493 (81.1) *	302 (61.3)	191 (38.7)	
Complementary feeding before 6 months				0.291
Yes	456 (75.0) *	272 (59.6)	184 (40.4)	
No	152(25.0) *	98 (64.5)	54 (35.5)	
Bottle feeding in the first year of life				0.852
Yes	131 (21.8) *	81 (61.8)	50 (38.2)	
No	471 (78.2) *	287 (60.9)	184 (39.1)	
Use of a pacifier before 6 weeks old				0.899
Never used	442 (72.9) *	267 (60.4)	175 (39.6)	
Used ≥6 weeks	60 (9.9) *	38 (63.3)	22 (36.7)	
Used <6 week	104 (17.2) *	64 (61.5)	40 (38.3)	

^1^ Differences from the total number of participants observed in some variables are due to missing information in that variable; ^2^ MW: minimum wage; ^3^ EBF: exclusive breastfeeding; EBFme: exclusive breastfeeding with early formula use); MBF: mixed breastfeeding. * Percentage differences that are statistically different (*p* < 0.05).

**Table 2:** Risk of continued breastfeeding interruption at 12, 18, and 24 months according to maternal sociodemographic characteristics, prenatal care, and child characteristics. Rio Branco, Acre, 2015–2017.

Variable	Median Time	Risk of Weaning from 6–24 Months			Wilcoxon–Gehan
	Days	12	18	24	*p*-Value
Global		19%	65%	71%	
Maternal age (years)					0.794
≤19	295	18%	28%	75%	
20–34	300	19%	65%	71%	
≥35	315	21%	60%	66%	
Skin color					0.377
White	304	19%	65%	69%	
Brown	302	18%	64%	70%	
Others	270	22%	78%	82%	
Maternal education					0.463
Up to 8 years	293	21%	67%	72%	
8 years or more	304	18%	64%	71%	
Marital status					0,477
Without partner	302	17%	65%	72%	
With partner	292	27%	64%	66%	
Income level ^1^					0.702
≤1 MW	299	21%	65%	68%	
1 to 3 MW	304	18%	64%	70%	
>3 MW	296	18%	68%	67%	
Bolsa Família Program					0.539
Yes	299	18%	63%	70%	
No	306	19%	66%	71%	
Socioeconomic class					0.168
A and B	288	26%	66%	74%	
C, D and E	303	17%	65%	70%	
Smoking					0.994
Yes	305	22%	62%	64%	
No	301	18%	65%	71%	
Alcohol					0.004
Yes	266	25%	77%	81%	
No	308	18%	63%	69%	
Prenatal care (visits)					0.444
<6	307	17%	64%	68%	
≥6	300	19%	65%	72%	
Prenatal care sector					0.080
Public	304	18%	65%	69%	
Private	277	27%	70%	81%	
Planned pregnancy					0.070
Yes	311	17%	62%	69%	
No	294	20%	67%	73%	
Prenatal breastfeeding guidance					0.171
Yes	313	18%	61%	68%	
No	289	20%	70%	73%	
Birth history					0.480
Primiparous	303	17%	65%	72%	
Multiparous	299	20%	65%	70%	
Number of children					0.698
1	303	17%	65%	72%	
2–3	292	19%	69%	74%	
≥4	309	21%	61%	67%	
Received formula in the maternity ward					0.669
Yes	307	21%	62%	77%	
No	299	18%	64%	72%	
Breastfeeding support in the maternity ward					0.140
Yes	306	18%	63%	68%	
No	288	20%	70%	78%	
Self-reported postpartum depression					0.372
Yes	307	16%	64%	66%	
No	299	19%	66%	72%	
Breastfeeding at hospital discharge ^2^					0.487
EBF	298	19%	66%	72%	
EBFme	318	20%	59%	65%	
MBF	324	10%	60%	70%	
Stopped working due to the baby’s birth					0.008
Yes	285	23%	69%	75%	
No	317	14%	61%	67%	
Mother’s desire for breastfeeding duration					0.432
<6 months	313	12%	63%	69%	
≥6 months	299	20%	65%	71%	
Baby’s sex					0.429
Female	296	20%	67%	71%	
Male	307	18%	63%	70%	
Positive paternal involvement in breastfeeding					0.500
Yes	297	18%	67%	72%	
No	322	22%	52%	64%	
Prematurity					0.340
Yes	323	16%	59%	63%	
No	299	19%	66%	72%	
Childcare follow-up					0.734
≤7 days	301	19%	65%	70%	
>7 days	302	18%	66%	73%	
Breastfeeding in the first hour of life					0.309
Yes	304	16%	365%	70%	
No	294	22%	66%	73%	
Cross-nursing					0.805
Yes	306	16%	64%	70%	
No	299	18%	65%	71%	
Complementary feeding before 6 months					0.248
Yes	296	20%	66%	72%	
No	315	16%	61%	68%	
Bottle feeding in the first year of life					0.001
Yes	265	28%	74%	81%	
No	311	16%	62%	68%	
Use of a pacifier before 6 weeks old					0.000
Never used	316	11%	61%	67%	
Used <6 weeks	247	32%	81%	86%	
Used ≥6 weeks	289	22%	68%	78%	

^1^ MW: minimum wage; ^2^ EBF: exclusive breastfeeding; EBFme: exclusive breastfeeding with early formula use); MBF: mixed breastfeeding.

**Table 3:** Factors associated with the risk of continued breastfeeding interruption between 6 and 24 months of age in Rio Branco, Acre, 2015–2017.

Variable	Proximal Model		Intermediate Model ^2^		Distal Model ^3^	
	HR	95CI%	HR	95CI%	HR	95CI%
Maternal age (years) ^1^	0.99	(0.97–1.00)	0.98	(0.97–1.00)	0.98	(0.97–1.00)
Baby’s sex						
Male	1	-	1	-	1	-
Femal	1.07	(0.88–1.29)	1.09	(0.88–1.34)	1.12	(0.91–1.38)
Alcohol						
Yes	1.32	(1.03–1.69)	1.33	(1.01–1.75)	1.27	(0.96–1.68)
No	1	-	1	-	1	-
Prenatal care sector						
Public	1		1	-	1	-
Private	1.34	(1.02–1.75)	1.19	(0.89–1.60)	1.07	(0.79–1.44)
Stopped working due to the baby’s birth						
Yes	-	-	1	-	1	-
No	-	-	0.80	(0.64–0.99)	0.78	(0.62–0.97)
Use of a pacifier before 6 weeks old						
Never used	-	-	-	-	1	-
Used ≥6 weeks	-	-	-	-	1.27	(0.90–1.78)
Used <6 weeks					1.62	(1.24–2.11)
Bottle feeding in the first year of life						
Yes	-	-	-	-	1.41	(1.11–1.78)
No	-	-	-	-	1	-

^1^ Data analyzed continuously. ^2^ Adjusted for maternal age and baby’s sex. ^3^ Adjusted for maternal age, baby’s sex, maternal alcohol consumption during pregnancy, prenatal care sector, maternal return to work after the baby’s birth, pacifier use before 6 weeks of age, and bottle use in the first year of life.

## Data Availability

The data presented in this study are available on request from the corresponding author as they are confidential and were obtained under informed consent.

## Appendix A

**Variables**	**Included in the Analysis**		
	**No**	**Yes**	***p*-Value**
	**n (%)**	**n (%)**	
Maternal age (years)			
≤19	49 (8.1)	73 (12)	
20–34	160 (26.3)	239 (39.3)	
≥35	29 (4.8)	58 (9.5)	0.487
Skin color			
White	23 (3.8)	31 (5.0)	
Brown	191 (31.4)	323 (53.1)	
Others	24 (3.9)	16 (2.6)	0.015
Maternal education			
Up to 8 years	72 (11.8)	87 (14.4)	
8 years or more	166 (27.3)	283 (46.5)	0.065
Marital status			
Without partner	32 (5.3)	56 (9.2)	
With partner	205 (33.8)	314 (51.7)	0.577
Income level ^1^			
≤1 MW	51 (9.2)	66 (11.9)	
1 to 3 MW	126 (22.7)	187 (33.8)	
>3 MW	43 (7.8)	81 (14.6)	0.352
Bolsa Família Program			
Yes	61 (10)	92 (15.2)	
No	177 (29.2)	277 (45.6)	0.847
Socioeconomic class			
A and B	42 (7.0)	80 (13.3)	
C, D and E	195 (32.3)	286 (47.4)	0.217
Smoking			
Yes	17 (2.8)	28 (4.6)	
No	218 (36.1)	341 (56.5)	0.872
Alcohol			
Yes	46 (7.6)	49 (8.1)	
No	192 (31.6)	321 (52.7)	0.044
Bottle feeding in the first year of life			
Yes	50 (8.3)	81 (13.5)	
No	184 (30.5)	287 (47.7)	0.468
Use of a pacifier before 6 weeks old			
Never used	175 (73.8)	267 (72.4)	
Used ≥6 weeks	40 (16.9)	64 (17.3)	
Used <6 weeks	22 (9.3)	38 (10.3)	0.899
^1^ MW: minimum wage.			

## Appendix B

Unadjusted Cox regression analysis of factors associated with the risk of continued breastfeeding interruption between 6 and 24 months of age in Rio Branco, Acre, 2015–2017.

**Variables**	**HR**	**95%CI**	***p*-Value**
Maternal age (years)			0.668
≤19	1.16	0.83–1.61	
20–34	1.07	0.81–1.43	
≥35	1	-	
Skin color			0.410
White	0.79	0.55–1.13	
Brown	1	-	
Others	0.75	0.47–1.20	
Maternal education			0.709
Up to 8 years	1.04	0.84–1.29	
8 years or more	1	-	
Marital status			0.950
Without partner	1	-	
With partner	1.00	0.78–1.29	
Income level ^1^			0.497
≤1 MW	0.86	0.64–1.16	
1 to 3 MW	0.87	0.68–1.10	
>3 MW	1	-	
Bolsa Família program			0.623
Yes	1	-	
No	1.05	0.84–1.31	
Socioeconomic class			0.271
A and B	1	-	
C, D and E	1.14	0.90–1.43	
Smoking			0.624
Yes	0.91	0.62–1.32	
No	1	-	
Alcohol			0.015 *
Yes	1.35	1.06–1.73	
No	1	-	
Prenatal care (visits)			0.459
<6	0.92	0.73–1.14	
≥6	1	-	
Prenatal care sector			0.058 *
Public	1	-	
Private	1.28	0.99–1.67	
Planned pregnancy			0.238
Yes	1		
No	1.12	0.92–1.36	
Prenatal breastfeeding guidance			0.245
Yes	1	-	
No	1.13	0.91–1.40	
Birth history			0.916
Primiparous	0.99	0.81–1.20	
Multiparous	1	-	
Number of children			0.770
1	1	-	
2–3	1.05	0.84–1.32	
≥4	0.96	0.76–1.21	
Received formula in the maternity ward			0.619
Yes	0.92	0,69–1.24	
No	1	-	
Breastfeeding support in the maternity ward			0.084 *
Yes	1	-	
No	1.19	0.97–1.47	
Self-reported postpartum depression			0.243
Yes	0.86	0.67–1.10	
No	1	-	
Breastfeeding at hospital discharge ^2^			0.657
EBF	1	-	
EBFme	0.88	0.64–1.21	
MBF	0.85	0.50–1.45	
Stopped working due to the baby’s birth			0.074 *
Yes	1	-	
No	0.706	0.48–1.03	
Mother’s desire for breastfeeding duration			0.644
<6 months	1.04	0.86–1.26	
≥6 months	1	-	
Baby’s sex			0.796
Female	1	-	
Male	0.95	0.67–1.35	
Positive paternal involvement in breastfeeding			0.419
Yes	1	-	
No	0.89	0.68–1.17	
Prematurity			0.303
Yes	0.82	0.57–1.18	
No	1	-	
Childcare follow-up			0.606
≤7 days	1	-	
>7 days	0.94	0.74–1.18	
Breastfeeding in the first hour of life			0.361
Yes	1	-	
No	1.09	0.90–1.32	
Cross-nursing			0.888
Yes	1.01	0.79–1.29	
No	1	-	
Complementary feeding before 6 months			0.319
Yes	1.11	0.89–1.39	
No	1	-	
Bottle feeding in the first year of life			0.003 *
Yes	1.40	1.12–1.74	
No	1	-	
Use of a pacifier before 6 weeks old			0.000 *
Never used	1		
Used ≥6 weeks	1.32	0.97–1.79	
Used <6 week	1.67	1.32–2.12	
* *p*-value < 0.20. ^1^ MW: minimum wage; ^2^ EBF: exclusive breastfeeding; EBFme: exclusive breastfeeding with early formula use; MBF: mixed breastfeeding.			
